# Protein tyrosine phosphatases in cardiac physiology and pathophysiology

**DOI:** 10.1007/s10741-018-9676-1

**Published:** 2018-02-02

**Authors:** Fallou Wade, Karim Belhaj, Coralie Poizat

**Affiliations:** 1 Cardiovascular Research Program, King Faisal Specialist Hospital and Research Centre, PO Box 3354, Riyadh, 11211 Saudi Arabia; 2 College of Medicine and Health Sciences, Al-Faisal University, Riyadh, 11211 Saudi Arabia; 3 Biology Department, San Diego State University, San Diego, CA 92182 USA

**Keywords:** Protein tyrosine phosphatase, PTP, Cardiac hypertrophy, Heart failure, Small-molecule inhibitor

## Abstract

More than any other organ, the heart is particularly sensitive to gene expression deregulation, often leading in the long run to impaired contractile performances and excessive fibrosis deposition progressing to heart failure. Recent investigations provide evidences that the protein phosphatases (PPs), as their counterpart protein kinases, are important regulators of cardiac physiology and development. Two main groups, the protein serine/threonine phosphatases and the protein tyrosine phosphatases (PTPs), constitute the PPs family. Here, we provide an overview of the role of PTP subfamily in the development of the heart and in cardiac pathophysiology. Based on recent in silico studies, we highlight the importance of PTPs as therapeutic targets for the development of new drugs to restore PTPs signaling in the early and late events of heart failure.

## Introduction

Cardiac physiology is heavily dependent on the intracellular signaling balance between protein kinases (PKs) and their counterparts, the protein phosphatases (PPs). Not surprisingly, the deregulation of such balance in the adult heart leads in most cases to heart dysfunction associated with impaired contractile performances and fibrosis deposition often leading to heart failure [1–4]. In the past decade, the signaling cascades of numerous PKs and their partners in mediating pathological cardiac hypertrophy and heart failure have been extensively studied and reviewed from investigations performed in cellular models and in genetically modified mice (reviewed in [5–7]). However, proportionally, the role of PPs in the development of myocardial disorders has been much less documented [8, 9]. This is mostly due to historical reasons as PPs were discovered 10 years after PKs [10, 11]. Over the past decades however, it has become clear that PPs are specific regulators that play active roles in coordinating with PKs the regulation of many physiological processes. The PPs can be divided into two main groups, which are the protein serine/threonine phosphatases (PPPs) and the protein tyrosine phosphatases (PTPs). The PPPs constitute the majority of the PPs and target phosphoproteins on their serine and/or threonine residues. The three most documented PPPs in the heart are protein phosphatase 1 (PP1), protein phosphatase 2A (PP2A), and protein phosphatase 2B (PP2B) also known as calcineurin. Mechanisms by which PPPs regulate Ca^2+^ homeostasis and cardiac function are well documented in human and animal models of heart failure [12–14]. Since PPPs have been the topic of many excellent reviews, they will not be discussed in this review [9, 15, 16]. The PTPs dephosphorylate tyrosyl residues in proteins [17, 18] with the majority of PTPs being active enzymes and as abundant as protein tyrosine kinases (PTKs) [10]. Here, we summarize the role of the PTPs in heart development and cardiovascular diseases, particularly their effect on cardiac hypertrophy and how their dysregulation progresses to heart failure. Finally, we discuss the inhibitors of PTPs and their therapeutic potential for the treatment of heart disease in human.

## Protein tyrosine phosphatase family and substrate specificity

In human, more than 100 genes encode for the PTPs among which 81 are active phosphatases [10, 19, 20]. The PTPs constitute a large family of enzymes, many of which harbor a transmembrane domain and a variable ectodomain (for review, see [21–23]). All PTPs share a common signature motif (HCXXGXXR) responsible for the enzyme activity. The PTP superfamily can be divided into four classes based on their cellular localization/catalytic domains: the receptor-like PTPs (rPTPs), the non-receptor PTPs (nrPTPs), the low molecular weight PTP (LMWPTP), and the VH-1 and CDC-25 groups [10, 24–27]. A different classification of PTPs exists based on their amino acid sequence and catalytic domain, which groups them into four classes (reviewed in [10]). Class I includes rPTPs and nrPTPs also known as “classical” pTyr-specific PTPs. These comprise 38 PTPs, and VH1-like “dual-specificity” PTPs (DUSPs) which are very divers with 61 members able to dephosphorylate pTyr and pSer/pThr residues. Class II PTP has a single member, the LMWPTP that targets substrates specifically on their Tyr residues. Class III PTPs include three “dual-specific” Cdc25 enzymes, while class IV is represented by Eya proteins with pTyr or dual pTyr/pSer activity.

Tyrosine phosphorylation process mediates most if not all cell signaling processes including growth, differentiation, survival, and death [28]. In the early 1990s, the PTPs were mainly considered as housekeepers or passive players in the cell [10, 11]. After considerable efforts in the field, it is now recognized that PTPs are critical regulators of cell signaling. Deregulation of their expression or activity can compromise cell physiology and hence lead to diseases [29]. The importance of PTPs in regulating signaling pathways was first illustrated by the discovery of CD25, (a DUSP) and SHP2, which can positively regulate signaling by increasing the phosphorylated level of a tyrosyl site of PTK [30–33]. PTPs act as important regulators of tyrosine phosphorylation in many cell types including cardiac cells [34, 35].

## Role of PTPs in cardiac development and diseases

**Fig. 1:**
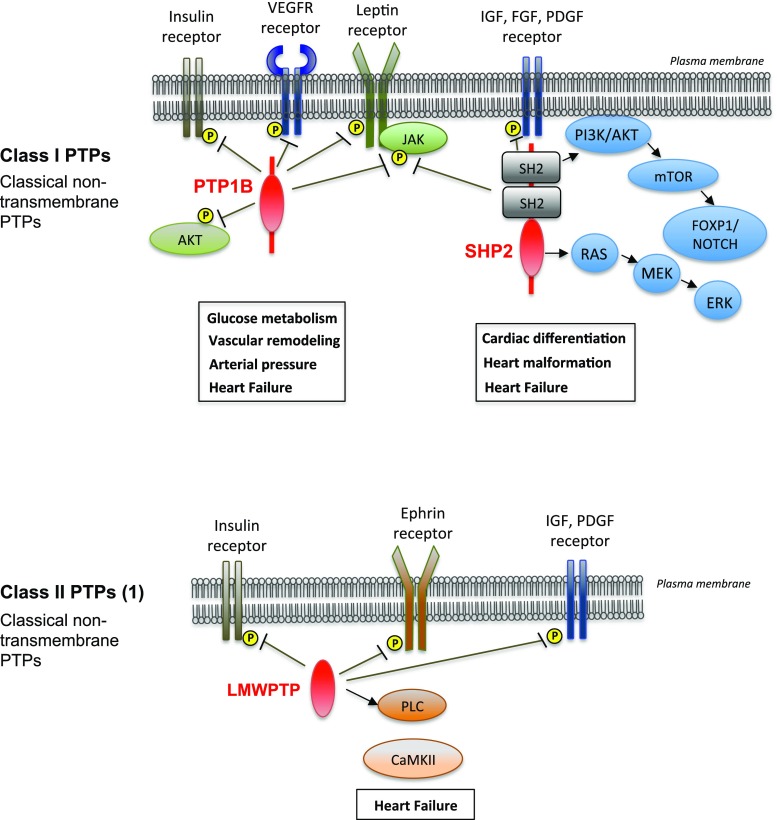
Protein tyrosine phosphatases (PTPs) playing a role in the cardiovascular system. Among the PTPs, three non-transmembrane PTPs have been described with roles in the cardiovascular system: protein tyrosine phosphatase 1B (PTP1B) and Scr-homology 2 domain containing phosphatase 2 (SHP2) belonging to class PTPs, and low molecular weight protein tyrosine phosphatase (LMWPTP), the sole member of class II PTPs

Out of the 107 human genes encoding PTPs, very few have been reported to have a role in the cardiovascular system. Up to this date, the PTPs implicated in cardiac development and disease include the protein-tyrosine phosphatase 1B **(**PTP1B), the Scr homology-2 (SH2) domain-bearing non-transmembrane protein tyrosine phosphatase (SHP2), and the LMWPTP (Fig. 1). Recent studies used genome-wide siRNA/shRNA screening and proteomics approach to identify novel roles and substrates of PTPs in human pathologies [36]. This powerful technology will be useful and adaptable for investigators to discover new PTPs implicated in human cardiovascular diseases. Below, we summarize mechanisms by which these PTPs regulate cardiac contractility and their role in heart development and disease (Table 1).

**Table 1:** Summary of PTPs implicated in cardiac development and disease

PTP	Animal model	Effect on the cardiovascular system	Comments	Reference
PTP1B (*PTPN1 gene*)	Global PTP1B deletion in Balb/c mice	PTP1B deficiency enhances the effects of leptin on arterial pressure	• Normal heart/body weight ratio, insulin, leptin, glucose, and triglyceride levels in fasting condition • Increased blood pressure at base line and after leptin infusion • Blunted response to phenylephrine • Reduction of aortic contraction after phenylephrine injection	[37]
	Global PTP1B deletion in Balb/c mice or PTP1B chemical inhibition	PTP1B deficiency protects against chronic heart failure after myocardial infarction	• No phenotype at baseline • Improvement of cardiac contractility and reduction of fibrosis and hypertrophy • Preserved endothelial function	[38]
	Endothelial-specific deletion of PTP1B in mice	PTP1B deletion in endothelium improves angiogenesis and protects against pressure overload induced heart failure	• Improved systolic function and reduced cardiac hypertrophy of PTP1B-deficient mice • Preserved capillary density, reduced apoptosis and fibrosis • Improved cardiac VEGF signaling	[39, 40]
	Global PTP1B deletion in Balb/c mice	PTP1B deletion enhances angiogenesis and arteriogenesis after myocardial infarction	• Increased capillary density • Increased VEGFR2 activation • Reduced diastolic dysfunction	[41]
SHP2 (*PTPN11 gene*)	*Xenopus* cardiac explants treated with the SPH2 inhibitor NSC-87877	SHP2 is required for the maintenance of cardiac progenitor cells	• Reduction of MHC expression, lack of early cardiac markers and of and pharyngeal mesoderm in cardiac progenitors • SHP2 controls Ca^2+^ transient and oscillation in fibroblasts and in cardiac myocytes • SHP2 is positioned downstream of FGF	[42]
	Genetic deletion of SHP2 exon 3 in mice (SHP2^Ex3−/−)^	Gain-of-function/Noonan syndrome SHP2 mutants increase Ca^2+^ oscillations and impair NFAT signaling in fibroblasts and cardiomyocytes	• SHP2 is required for Ca^2+^ oscillations in response to FGF-2 in fibroblasts and cardiomyocytes • Gain-of-function SHP2 mutants disrupt the Ca^2+^ oscillatory control of NFAT	[43]
	Cardiac specific deletion of SHP2	SHP2 deletion in cardiac muscle causes dilated cardiomyopathy with no hypertrophy	• Defective ERK/MAPK activity and hyper-activation of Rho signaling after agonist treatment of cardiomyocytes and pressure overload	[44]
	Overexpression of Noonan syndrome SHP2-Q79R in mice	Congenital heart defects are rescued by ERK1/2 activation	• Impaired cycling activity, ventricular non-compaction, and septal defects • Activation of ERK1/2	[45]
	Muscle specific deletion of SHP2	SHP2 deletion in skeletal muscle causes dilated cardiomyopathy, heart failure, and premature death	• Severe dilated cardiomyopathy resulting in heart failure and death • Associated with insulin resistance, glucose intolerance, and impaired glucose uptake in striated muscle cells • Upregulation of PI3K/Akt, ERK5, and STAT3 in cardiomyocytes	[46]
	Knockin SHP2-Y279C mutation in mice	Recapitulation of LEOPARD syndrome by expression of the SHP2-Y279C mutation. Cardiomyopathy reversed by rapamycin treatment	• Increased binding of SHP2 to IRS1 • Decreased SHP2 catalytic activity, and blunted ERK/MAPK activation and increased AKT and mTOR after agonist treatment	[18]
	Overexpression of SHP2-Q510E in mice	Early onset hypertrophic cardiomyopathy	• Impaired contractile function, thickening of the ventricular wall • Activation of AKT and mTOR • Rescue of cardiomyopathy by rapamycin treatment at the post-natal stage	[3]
	Expression of LEOPARD and NOON SYNDROM mutations in zebrafish	Impaired early heart development and function	• Activation of MAPK in embryos expressing the mutations	[47]
	Knockin SHP2-Y279C mutation in mice	Expression of SHP2-Y279C causes developmental defects and adult onset HCM in mice originating from the endocardium	• Reduced trabeculation and valvular hyperplasia in embryonic hearts expressing SHP2-Y279C and in the heart of mice with endothelial-specific but not myocardial-specific SHP2-Y279C expression • Endothelial-specific expression of SHP2-Y279C induces cardiac hypertrophy in the adult heart • Myocardial-specific SHP2-Y279C expression causes ventricular septal defects • Abnormal AKT activity and decreased forkhead box P1 (FOXP1)/FGF and NOTCH1/EPHB2 signaling	[48]
LMWPTP (*ACP1 gene*)	Global deletion of *ACP1* in mice	*ACP1* deletion protects against pathological cardiac stress	• Attenuated fibrosis and preserved cardiac contractility • Increased IR phosphorylation, PKA, and Ephrin expression • Reduced CaMKII expression	[4]

### PTP1B

The protein-tyrosine phosphatase 1B **(**PTP1B) is a non-transmembrane PTP with a wide tissue distribution and expressed mainly in the endoplasmic reticulum (ER) via its C-terminal domain [49]. PTP1B binds directly to PTK receptors including the insulin receptor (IR) and epidermal growth factor (EGF) receptor [50–53]. PTP1B is a key negative regulator of both insulin and leptin pathways ([54, 55] for review). Genetic deletion of PTP1B in mice results in insulin sensitivity and protects mice against high-fat diet-induced obesity [52, 56, 57]. High-fat diet-induced obesity increases the risk of hypertension and cardiovascular disorders, although the mechanism of action is unknown [58, 59]. Insulin resistance is strongly associated with oxidative stress, cardiac aging, and cardiomyocyte contractile dysfunction. Consistent with the effects of obesity and insulin on the cardiovascular system, PTP1B has emerged as a key regulator of obesity-induced cardiovascular disorders (recently reviewed in [39]).

Early studies using cardiac overexpression of antioxidant enzymes and dietary models documented reduced Akt expression and phosphorylation associated with increased PTP1B cardiac expression, suggesting a role of PTP1B in cardiac function [60, 61]. Enhancement of PTP1B was associated with impaired cardiac contractile and intracellular Ca^2+^ dysfunction [52, 60, 61]. As expected, PTP1B overexpression correlated with decreased phosphorylation of the IR (Tyr1146) and Akt after insulin stimulation in advanced cardiac aging and pre-diabetic insulin resistant hearts [60, 61]. Gomez and colleagues established the role of PTP1B in heart failure when they showed that mice with gene deletion or specific inhibition of PTP1B are protected against cardiac contractile dysfunction and heart failure after myocardial infarction. Improved heart function correlated with reduced fibrosis and hypertrophy while infarct size did not appear to change [38] (Table 1). In line with this study, recent findings showed that mice genetically lacking PTP1B resisted against chronic afterload-induced heart failure via a cardiac improvement of VEGF and angiogenesis signaling [39, 40]. Interestingly, eNOS, which modulates insulin secretion, was reduced in insulin resistant hearts or increased in PTP1B-deficient mouse hearts [38, 61]. Whether eNOS is directly associated with the insulin signaling is unclear, however the reduction of ROS generation alleviated insulin resistance-induced contractile dysfunction. PTP1B deletion enhanced capillary density and myocardial perfusion in mice 8-day post-myocardial infarction associated with increased VEGFR2 activity, although no reduction of infarct size was observed. This suggests that the beneficial effect of PTP1B ablation is mostly due to improved vascular remodeling possibly through nitric oxide (NO) [41]. Consistent with this, Panzhinskiy and collaborators reported that ER stress activates PTP1B via ROS-NFkB signaling resulting in insulin resistant in skeletal muscles under high-fat diet condition [52]. Also, endothelial dysfunction associated with diabetes did not occur in PTP1B-deficient mice mostly due to increased cyclooxygenase 2 expression [62]. Collectively, these studies are consistent with a protective role of PTP1B deletion against pathological cardiac and vascular remodeling. This is in contrast with the study by Belin de Chantemele and colleagues, where PTP1B knockout mice exhibited high blood pressure in response to leptin infusion [37]. This study indicates that PTP1B is a modulator of cardiovascular function through its capacity to negatively regulate leptin-induced hypertension possibly through the sympathetic nervous system. Overall, there is clear-cut evidence for a role of PTP1B in heart physiology and pathophysiology. More investigations are needed to define the underlying mechanisms by which PTP1B affects cardiovascular function. In this regard, a signaling of interest not yet elucidated is the alteration of Ca^2+^ handling through modulation of SERCA2a and NCX1 by PTP1B in the heart. Dysregulation of the sarco/endoplasmic reticulum Ca^2+^-ATPase (SERCA2a) and NCX1 expression impair cardiac contractility. In cancer, targeting of Ca^2+^ signaling has been proposed as an alternative therapy to treat human cancer patients [63]. The use of animal models lacking or overexpressing PTP1B and cellular models with genetic siRNA targeting of PTP1B could be a starting point to decipher the SERCA2a-NCX1-PTP1B axis in altered Ca^2+^ signaling-driven heart failure.

#### SHP2

The Scr homology-2 (SH2) domain-bearing non-transmembrane protein tyrosine phosphatase SHP2 also known as protein-tyrosine phosphatase non-receptor type 11 (PTPN11) or protein tyrosine phosphatase 2C (PTP-2C) is encoded by the *PTPN11* gene. SHP2 is ubiquitously expressed [64] and is important for the full activation of downstream partners (Ras/ERK/MEK) for most if not all the PTKs and cytokine receptors. SHP2 regulates important cellular events including differentiation, proliferation, and survival [64]. Key signaling pathways affected by SHP2 dysregulation include ERK1/2, insulin, AKT/GSK-3β, and mTOR pathways [3, 18, 44–46, 65] (Table 1). Not surprisingly, aberrant expression of SHP2 or changes within SHP2 activity are associated with human diseases and experimental animal models.

SHP2 is a key PTP required for early development. To avoid the embryonic lethality associated with SHP2 ablation, the role of SHP2 in early heart development was first addressed using *Xenopus* cardiac explants treated with the SHP-2-specific inhibitor NSC-87877 [42]. Results showed a reduction of myosin heavy chain expression, a lack of early cardiac markers of differentiation and of pharyngeal mesoderm. SHP2 interacted with FRS2 and that effect was associated with increased phosphorylation of SHP2 at both tyrosine 542 and 580. Collectively, this study positioned SHP2 downstream of FGF and showed that SHP2 is required for the maintenance of cardiac progenitors and survival in *Xenopus* embryonic hearts. Additional studies support the direct role of SHP2 in cell development and survival through the FGF signaling pathway [42, 66, 67]. Deletion of SHP2 in skeletal and cardiac muscle also causes cardiac dysfunction leading to dilated cardiomyopathy and premature death [46].

Germline mutations in SHP2 cause Noonan syndrome (NS) in human. This relatively common condition affects 1 in 1000–2000 children born with heart malformations including pulmonary valvular stenosis, septal defect, hypertrophic cardiomyopathy, and also abnormal facial characteristics and developmental delays [68, 69]. SHP2 mutations are also implicated in NS with multiple lentigines known as LEOPARD syndrome (LS). This rare genetic condition is associated with congenital heart malformations and also sensorineural deafness, growth retardation, and skin, craniofacial, and genital abnormalities [70, 71] (for reviews). Although NS and LS share common clinical features, SHP2 mutations are activating in NS due to increased phosphatase activity and inactivating in LS because of an inhibition of the catalytic activity of the phosphatase [72, 73]. However, while the majority of NS mutations have a gain-of-function phenotype, there is also documentation that LS-causing mutations reduce SHP2 phosphatase activity but prolong substrate turnover to produce a loss-of-function phenotype [74].

The effects of NS- and LS-associated SHP2 mutations on cardiac morphogenesis can be recapitulated in mice and are due to increased MAPK signaling. Indeed, independent investigations demonstrated that both Q79R SHP2 gain of function and lack of SHP2-induced hyperactivation of ERK1/2 and RhoA signaling, leading to impaired heart function and dilated cardiomyopathy [44, 45]. Expression of the LS mutant Q510E causing severe hypertrophic cardiomyopathy in infants inhibits the differentiation of P19CL6 cells in cardiomyocytes mostly due to increased Akt/GSK3β/β-catenin activity [3, 18, 44–46, 65], and induces hypertrophic cardiomyopathy in mice through mTOR pathway [3].

Gain-of-function SHP2 mutants (R465M, E76A, D61G) enhanced Ca^2+^ response in cardiomyocytes through RTKs mediated Ca^2+^ signaling pathway but not upon activation of G protein-coupled receptor [43], further supporting the requirement of SHP2 in the activation of most RTK signaling. Consistent with these findings, more recent data indicate that NS and LS SHP2 variants significantly enhanced ERK activity, which partly mediated defective early cardiac development in zebrafish [47]. Furthermore, expression of Shp2-Y279C, a mutation causing LS in human, recapitulated the phenotypic abnormalities seen in LS patients with signs of hypertrophic cardiomyopathy progressing to dilated cardiomyopathy and enhanced interaction of Shp2 with IRS1, and increased Akt/mTOR activity. These cardiac defects were totally reversed by treatment with the mTOR inhibitor rapamycin [18]. The developmental defects and adult-onset hypertrophic cardiomyopathy in Shp2-Y279C mutant mice correlated with increased AKT activity, inhibition of FOXP1/NOTCH1 pathways, and upregulation of NFAT activity. Dysregulated signaling originated from the endocardium indicating a reciprocal cross-talk between the endocardium and the myocardium, which is essential for heart development [48]. Such non-cell-autonomous mechanism was also triggered by overexpression of the related transcription enhancer factor-1 RTEF1 in endothelial cells, which induced cardiac hypertrophy in response to aortic constriction through an increase of VEGFB protein level [75]. Although studies of Lauriol and colleagues did not reveal enhanced expression of VEGFB mRNA level, they did not exclude a possible implication of SHP2 in NSML cardiac hypertrophy that employs this RTEF1-VEGF signaling mechanism [48].

#### LMWPTP

LMWPTP is a class 2 cys-based PTP encoded by the *ACP1* gene located on the short arm of chromosome 2 (2p25) in the human genome and widely distributed within various tissues and organs including the heart [10, 76, 77]. LMWPTP controls a number of essential processes in mammalian cell physiology [10]. This 18 kDa protein tyrosine phosphatase has three isoforms generated by alternative splicing. The first two isoforms produce functional proteins while the third isoform is considered a pseudogene [76, 78–80]. A decrease of both LMWPTP isoforms leads to increase in phosphorylation of the insulin receptor, Akt and PI3-K activity in the liver [81].

LMWPTP has five tyrosine residues and can be phosphorylated by tyrosine kinases such as V-src, Lck, and Fyn. Depending on the tyrosine residue phosphorylated, LMWPTP has different phenotypes. The phosphorylation of Tyr^131^ residue leads to a 25-fold increase of LMWPTP level while the phosphorylation of Tyr^132^ mediates the recruitment of Grb2 protein [82]. On the other hand, like other PTPs, LMWPTP can be dephosphorylated by tyrosine phosphatases, and can by itself dephosphorylate various tyrosine kinases and their respective substrates [24, 76].

Studies suggested that its overexpression causes increased dephosphorylation of phosphotyrosine, which may repress tyrosine kinase oncogene malignant transformation and growth factor receptor signaling [10]. LMWPTP also modulates the JAK-STAT pathway by binding and dephosphorylating STAT5 [83]. Furthermore, LMWPTP oxidation prevents dephosphorylation and inactivation of STAT2 and JAK5 [83]. In addition, LMWPTP is regulated by ROS mediated oxidation [84]. LMWPTP tyrosine residues can be oxidized by exogenous oxidative stress by glucose oxidase or sodium pervanadate in vivo [24]. Alteration of LMWPTP levels causes a reduction in enzymatic binding, glycolysis, and erythrocyte plasticity in T cell signaling [76]. Furthermore, an increase in LMWPTP levels was associated with protection against several conditions such as allergy, asthma, and abortion [76].

LMWPTP also acts as a negative regulator of EphA2 tyrosine phosphorylation, which regulates tumor cell growth and survival [83, 85]. LMWPTP affects cellular proliferation through reduction of FGFR tyrosine phosphorylation [24, 82, 86] and modulates PDGF expression through its phosphatase activity [84]. Overexpression of LMWPTP markedly decreases cell growth rate as a secondary effect of PDGF reduction [24]. LMWPTP also acts as a negative regulator of insulin signaling as shown by the improved glucose and insulin tolerance of diet-induced obese mice injected with an LMWPTP antisense oligonucleotide [81].

Examination of genetic variations in the *ACP1* gene revealed the presence of single-nucleotide polymorphisms (SNPs) that alter the enzyme activity and the ratio of the two main protein isoforms LMPTP-A and LMPTP-B [87]. Association studies in various populations indicated a role of *ACP1* in metabolic syndrome and coronary artery disease [88–91]. In particular, the hypertrophic response of the myocardial wall is regulated by LMWPTP through activation of growth factors such as PDGF, IGF1, and insulin [76]. Moreover, increased LMWPTP activity reduces the metabolic rate and subsequently enhanced the demand of the hypertrophic response [76]. Consistent with this report, deletion of LMWPTP in mice confers a cardio-protective phenotype after long-term pressure overload hypertrophy [4]. The striking reduction of fibrosis and sustained cardiac function of LMWPTP knockout mice subjected to pathological stress are associated with upregulation of fetal cardiac genes, increased insulin receptor phosphorylation, and inactivation of Gαq/11/PLCβ/CaMKII pathways. LMWPTP levels are also high in the fetal murine heart, reduced in the post-natal heart, and increased in patients with end-stage heart failure indicating that LMWPTP is a positive regulator of pathological cardiac hypertrophy [4].

## PTP inhibitors as potential new therapeutic for cardiac diseases

PTPs play a central role in modulating physiological cardiac development [35]. Hyper-activation of the catalytic domain of PTPs initiates cardiomyopathy through the deregulation of cellular processes like proliferation [43]. Therefore, the implication of PTPs in the development of cardiomyopathies supports PTPs as potential targets for signaling-based therapeutics for these diseases. Two decades ago, vanadate and pervanadate were widely used to inhibit PTPs including PTP1B [92] (Table 2). This inhibition revealed potent and selective to PTPs but unfortunately non-specific. Subsequently, several small molecules targeting PTPs have been developed or tested with the hope to treat human pathologies including heart failure, diabetes, and cancers. Among those, vanadyl sulfate protected against ischemia-reperfusion in rats via increased FLIP and decreased Fas ligand and Bim expression secondary to AKT activation [93]. However, when tested in diabetic patients, vanadyl sulfate altered the expression of proteins involved in early insulin signaling in skeletal muscle, with no effect on protein phosphatase activity [94]. Endothelial dysfunction of peripheral arteries after short-term ischemia-induced heart failure was improved in mice after administration of AS279, AS098, and AS713 PTP1B inhibitors [95]. Compound inhibitors of PTP1B have being developed in academic laboratories and by the pharmaceutical industry and tested in animal models of obesity. Some like trodusquemine and ertiprotafib have reached phase 2 clinical trials to treat obesity and diabetes, although ertiprotafib was discontinued for lack of efficacy [55, 99]. It remains to be seen whether PTP1B inhibition has long-term beneficial effects for the treatment of cardiovascular disorders such as congestive heart failure.

**Table 2:** Inhibitors of PTPs with effects in the cardiovascular system

Inhibitor	Targeted PTP	Effect on cardiovascular system	Model	Advantages	Disadvantages	Reference
Vanadate	PTPs			• Selective	• Non-specific	[92]
Vanadyl sulfate	PTPs	Cardioprotection against ischemia reperfusion	• Coronary occlusion in rats	• Reduction of infarct size and of left ventricular end diastolic pressure • Improvement of left ventricular developed pressure and contractility • Inhibition of apoptosis • Increased FLIP expression and decreased Fas ligand and Bim expression via activation of Akt	• Gastrointestinal side effects	[38, 93]
Vanadyl sulfate	PTPs	Alteration of proteins involved in early insulin signaling in skeletal muscle. No global change in protein phosphatase activity	• Human with diabetes mellitus	• Increased basal levels of IR, Shc, and IRS-1 tyrosine phosphorylation and IRS-1 • No effect on insulin	• Gastrointestinal side effects at 150 and 300 mg	[94]
AS279, AS098, AS713	PTP1B and SHP2	Improved endothelial dysfunction in peripheral mesenteric arteries	• Mice with coronary ligation	• Selective inhibitors • Transient early eNOS phosphorylation and AKT phosphorylation	• Relatively good selectivity	[95]
AS279	PTP1B	Protects against chronic heart failure after myocardial infarction	• Mice	• Reduced adverse ventricular remodeling • Improved LV function and cardiac contractility • Reduced fibrosis and cardiac hypertrophy • Preserved endothelial function • No effect on glucose level		[38]
NSC-87877	SHP2	SHP2 is required for the maintenance of cardiac progenitor cells	• Xenopus heart explants	• Reduction of MHC expression, lack of early cardiac markers and of and pharyngeal mesoderm in cardiac progenitors • SHP2 controls Ca^2+^ transient and oscillation in fibroblasts and in cardiac myocytes • SHP2 is positioned downstream of FGF		[42]
PHPS1	SHP2	Inhibits cardiac hypertrophy induced by SHP2-Q510E and SHP2-Y279C mutations	• Cardiomyocytes and mice	• Normalization of the size of cardiomyocytes expressing SHP2-Q510A and SHP2-Y279C • Restoration of AKT and mTOR levels	• Specificity	[96]
Chromones	LMWPTP and PTP1B			• High activity		[97]
Compound 23	LMWPTP	Small-molecule inhibitor reverses high fat diet-induced obesity	• Mice	• Improves glucose tolerance • Increases IR phosphorylation in the liver • Orally bioavailable • Uncompetitive mode of action • High selectivity	• Not reported	[98]

Chromones, which are derivatives of benzopyran, are a class of highly active inhibitors of LMWPTP. They also have selective inhibition towards PTP1B [97]. The findings that LMWPTP plays a role in cancer and heart failure and alters insulin signaling prompted interest to develop inhibitors of LMWPTP with high activity. A series of compounds were discovered which inhibit LMWPTP and also PTP1B [97]. Recently, several novel small-molecule inhibitors of LMWPTP with potency and strong selectivity were discovered [100]. Also, a non-competitive small-molecule inhibitor of LMWPTP with high selectivity over other phosphatase with the ability to reverse high-fat diet-induced obesity has been reported [98]. This new inhibitor is viewed as promising for the treatment of human diseases including type 2 diabetes and heart failure. Numerous efforts have been made to identify inhibitors of SHP2 since missense mutations cause NS and LS in human. High-throughput screening identified a small molecular weight compound PHPS1 with high specificity and cell permeability [101]. PHPS1 inhibits SHP2 catalytic domain and was found to prevent the hypertrophic effect of mutant SHP2-Q510E in isolated cardiomyocytes and in mice expressing the mutant protein [96, 101]. Since the SHP2-Q510E mutation causes an aggressive form of LS in human, it would be of interest to test whether the PHPS1 inhibitor confers cardioprotective effects in clinical settings. Compound inhibitors of SHP2 have also been identified using a computational approach [102]. One compound #220–324 proved efficient in inhibiting SHP2-mediated signaling and proliferation of cancer cells, but whether it is an ideal inhibitor to treat the cardiomyopathy associated with SHP2 mutations remains to be seen.

## Challenges with PTP inhibitors

Although recent advances are encouraging, the use of PTP inhibitors faced significant technical challenges in the early days. These were mostly due to the lack of PTP inhibitors with both specificity and strong binding activity. This was in part due to the small size of PTPs in addition to sharing a common catalytic signature motif (HCXXGXXR) responsible for the enzyme activity. For instance, NSC-87877 inhibited both Shp2 and Shp1 in vitro with the same efficacy [103]. Another important challenge still remaining today is the need to optimize and develop safer PTP inhibitors for heart failure treatment avoiding undesirable effects, in particular, cardiotoxicity. Thus, solving these technical challenges is critical before PTP inhibitors enter extensive preclinical trials to treat human heart failure.

Complementary concepts from computer-aided drug design (CADD) to 3D QSAR/structure-based design have been used in experimental assays to identify potential drugs targeting the catalytic pocket of PTPs, ligand binding, and conformational change during inhibitor interactions [104–106]. Information on the 3D crystal structure on PTPs is a key step for in silico screening of abundant inhibitors available in databases. SHP2 and PTP-1B are shown to be “druggable” molecules for treating cardiac diseases and other pathologies. However, few 3D crystal structure PTPs are currently available, thus making the computer search approach to find PTP inhibitors difficult [107–109]. PTP-targeted therapy is still in its early phase, although novel drugs are emerging for cancer treatment [75, 110, 111]. Further testing is needed to determine whether these new compounds can be used for pharmacological treatment of cardiomyopathies.

## Conclusion

Growing experimental data support the role of PTPs as activators of cardiac diseases that operate through different mechanisms. This extensive work led to consider PTP family as drug targets to treat the diverse forms of cardiomyopathies. Few PTP inhibitors are known compared to the number of existing compounds for the treatment of such diseases. With the discovery of new compounds based on new screening strategies combined with the information on the 3D crystal structure on PTPs, one hopes that the design of drugs targeting PTPs will open a door of opportunity to treat human heart failure.

## List of abbreviations

AKT: Protein kinase B
CaMK: Ca^2+^/calmodulin-dependent protein kinase
eNOS: Endothelial nitric oxide synthase
ERK: Extracellular signal-regulated kinase
FGFR: Fibroblast growth factor receptor
FOXP1: Forkhead box protein P1
FRS2: Fibroblast growth factor receptor substrate 2
GSK-3: Glycogen synthase kinase 3
HCM: Hypertrophic cardiomyopathy
JAK2: Janus kinase 2
JAK-STAT: Janus kinase/signal transducers and activators of transcription
mTOR: Mammalian target of rapamycin
NFAT: Nuclear factor of activated T cells
NFkB: Nuclear factor-kappa B
NOTCH1: Notch homolog 1, translocation-associated (*Drosophila*)
P19CL6: Clone of the P19 mouse embryonal carcinoma cell line
PLC: Phospholipase C
PI3-K: Phosphoinositide 3-kinase
PDGF: Platelet-derived growth factor
RhoA: Ras homolog gene family, member A
ROS: Reactive oxygen species
RTKs: Receptor tyrosine kinases
